# Supplementary figures and images for: Mapping the semi-nested community structure of 3D chromosome contact networks
Source: PLoS Comput Biol. 2023 Jul 11;19(7):e1011185. doi: 10.1371/journal.pcbi.1011185 (PMC10361492; doi:10.1371/journal.pcbi.1011185)

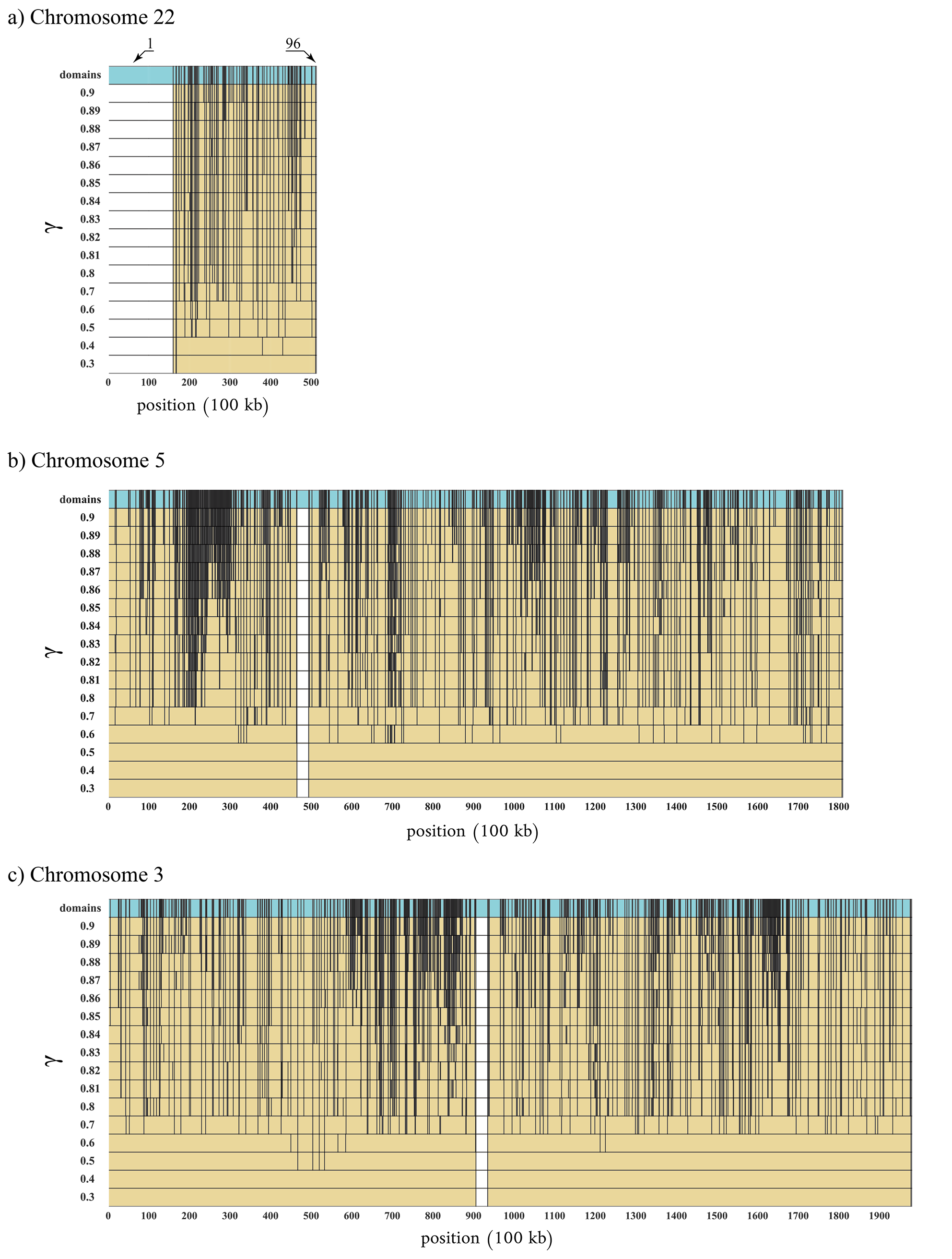

Supplement: S1 Fig — Each stripe represents a community partition for a single γ value. Within each stripe, vertical lines separate two adjacent DNA segments that belong to different communities. The white area shows the centromere. The top turquoise stripes in (a)—(c) show the domains. These are DNA segments that did not break across the shown γ range. In (a), we label two domains ‘1’ and ‘96,’ representing the first a last domain along chromosome 22. The domain IDs follow sequential order along the chromosome so that domain 2 is a linear neighbor of domains 1 and 3. (TIF) [file pcbi.1011185.s001.tif]

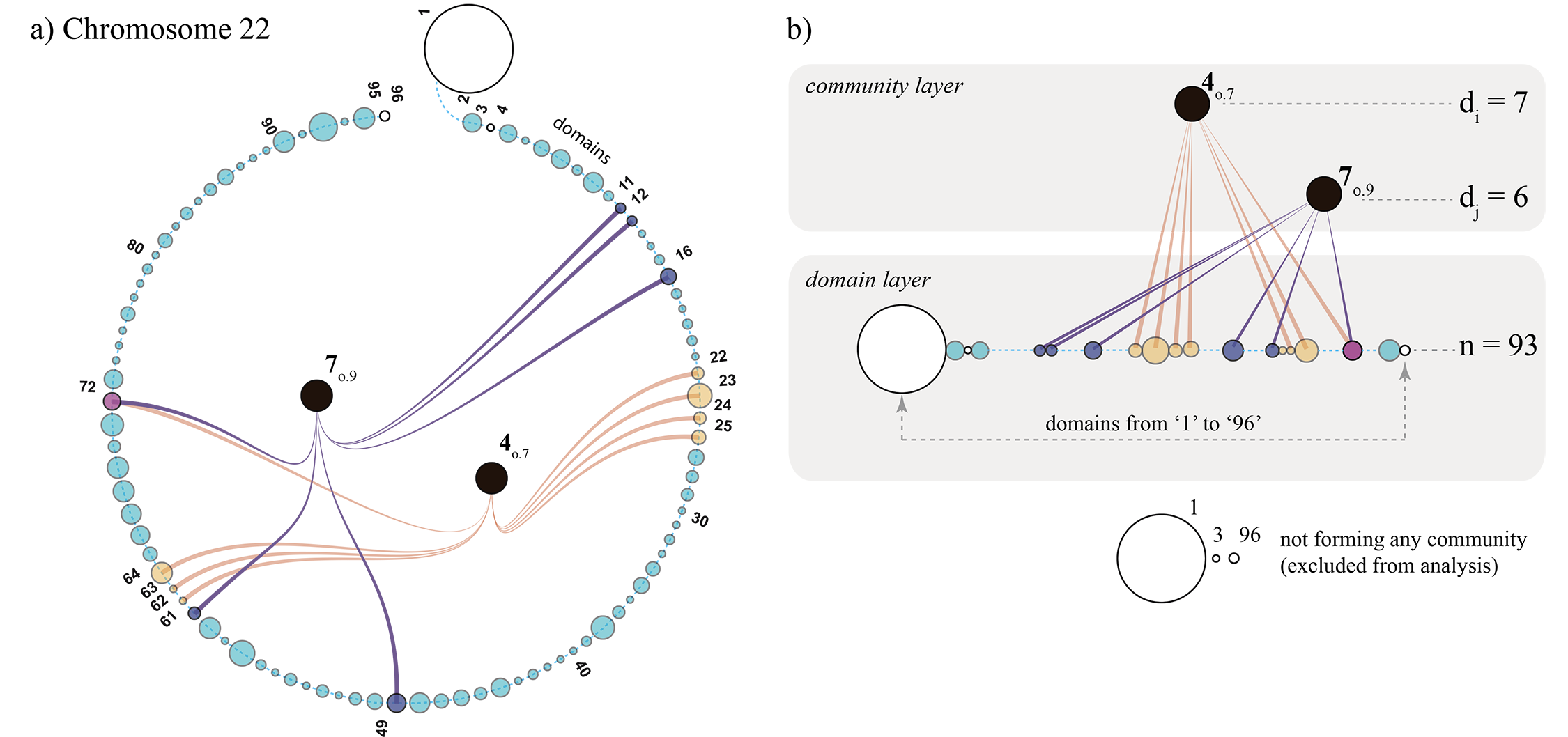

Supplement: S2 Fig — (a) We illustrate the domains as a chain of circles starting from ‘1’ and ending with ‘96.’ With colored links, we show domain memberships in two communities: 70.9 and 40.7. (b) Example of community pair overlap in a bipartite graph. The domains in the bottom layer connect to communities in the upper layer. White circles show domains that do not belong to any community (excluded from the nestedness analysis). (TIF) [file pcbi.1011185.s002.tif]

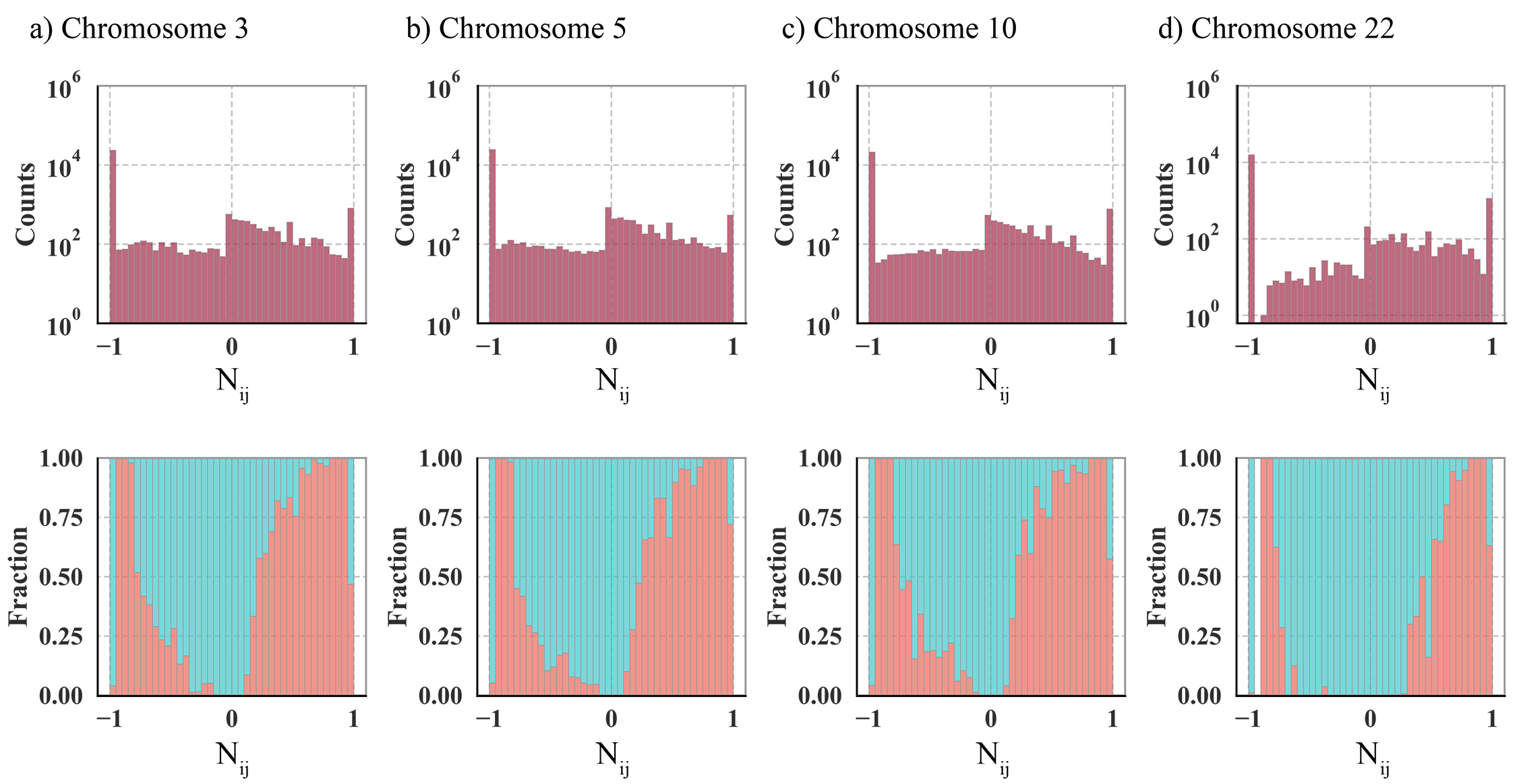

Supplement: S3 Fig — (top) Nestedness histograms for chromosomes 3, 5, 10, and 22. (bottom) The fraction of Nij scores that are significant (orange) or random (blue-green). (TIF) [file pcbi.1011185.s003.tif]

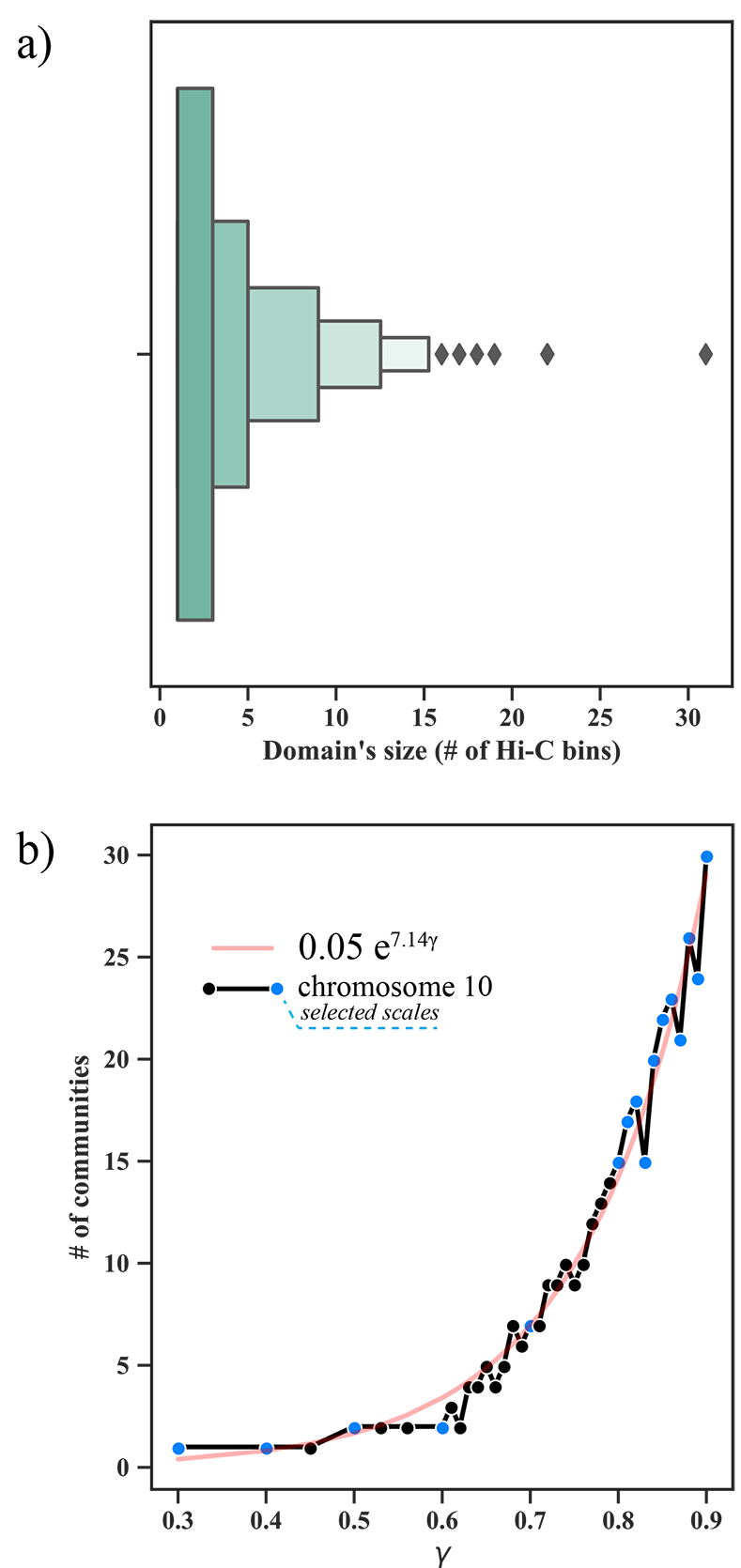

Supplement: S4 Fig — (a) Letter-value plot showing the size distribution of irreducible domains. The domain sizes vary between ∼1 − 30 Hi-C bins. The median domain size is 1 Hi-C bin (100 kb). (b) The scale-dependent number of communities (defined by γ). The number of communities grows exponentially with γ (red). (TIF) [file pcbi.1011185.s004.tif]

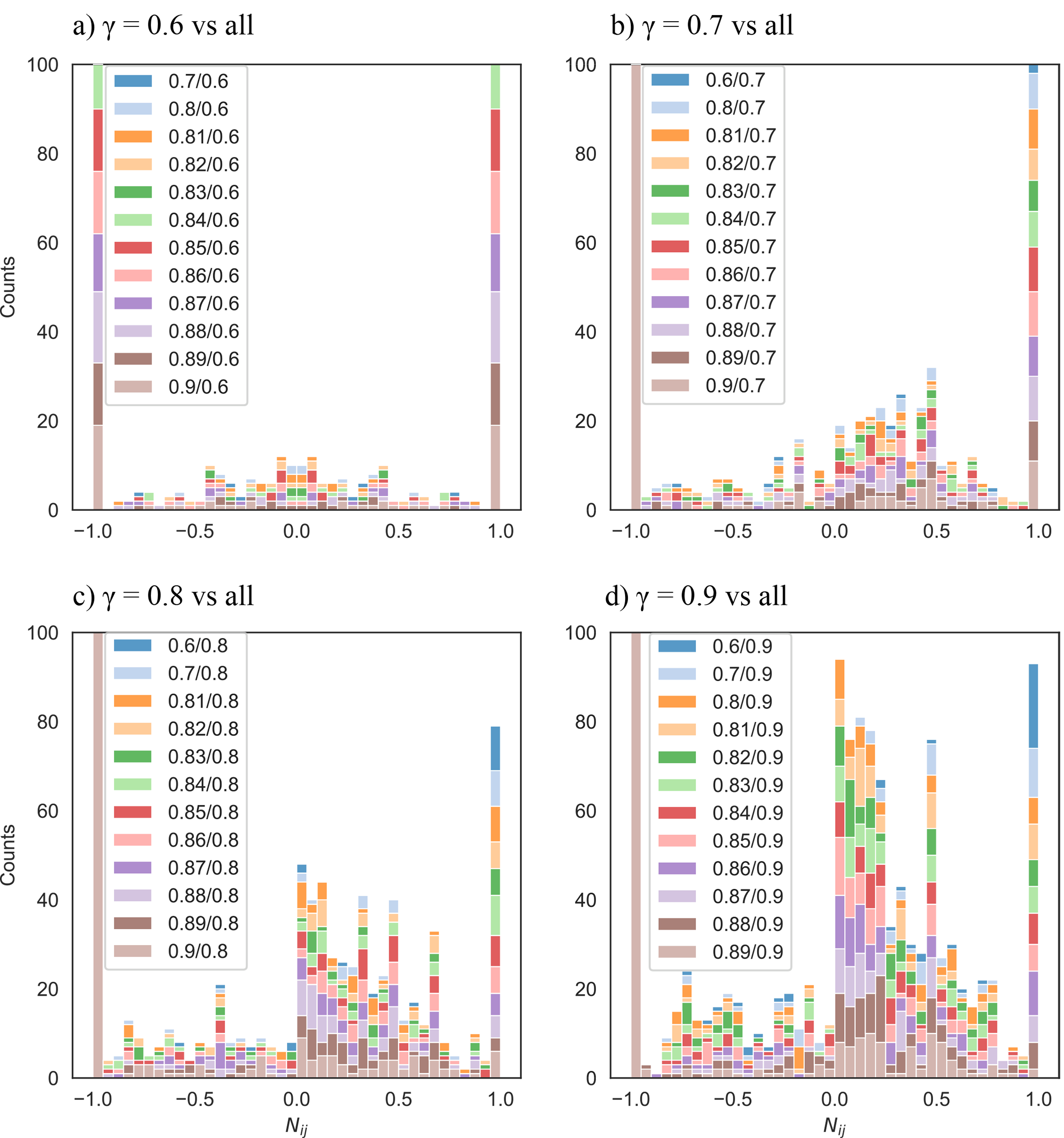

Supplement: S5 Fig — To better illustrate range −1 < Nij < 1, we truncate the bars at Nij ± 1 if their counts exceed 100. a) pairs between γ = 0.6 and all other γ. Cross-scale interaction between communities found at γ = 0.6 and all other communities are fully nested, fully segregated, but also their nestedness is close to random (−0.5 < Nij < 0.5); b) for γ = 0.7 and all other γ. The distribution changes for Nij ∼ 0), showing that communities tend to be more nested (more counts in the range: 0 < Nij < 0.5); c) and d) for γ = 0.8 and 0.9 versus all other γ. We observe that distribution peaks near Nij = 0 when comparing to other distributions. (TIF) [file pcbi.1011185.s005.tif]

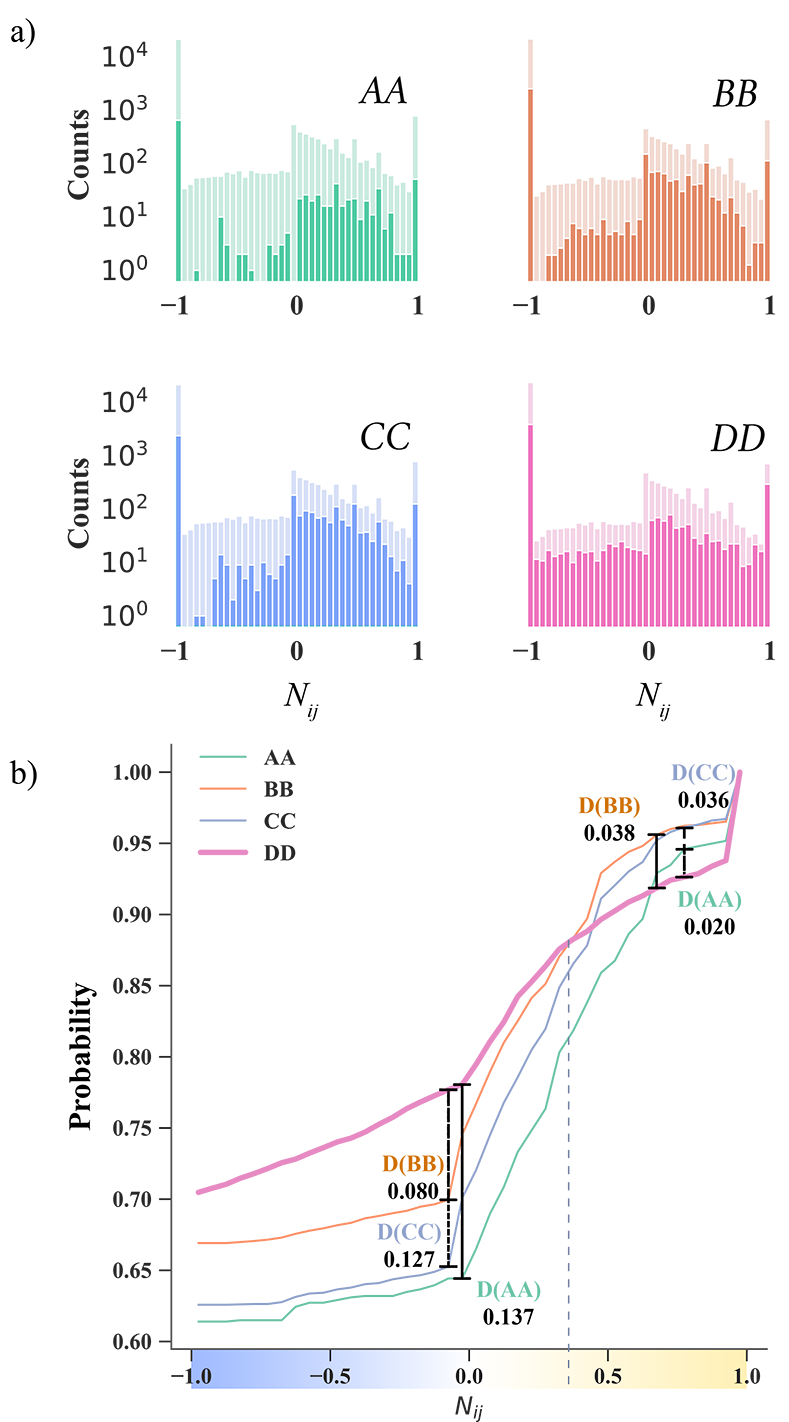

Supplement: S6 Fig — (a) Nestedness distributions (Nij) between communities of the same type (either A, B, C, or D). (b) CDF of Nij distributions. The vertical bars indicate the Kolmogorov-Smirnov distance between ‘DD’ distribution (thick pink line) and all others. (TIF) [file pcbi.1011185.s006.tif]

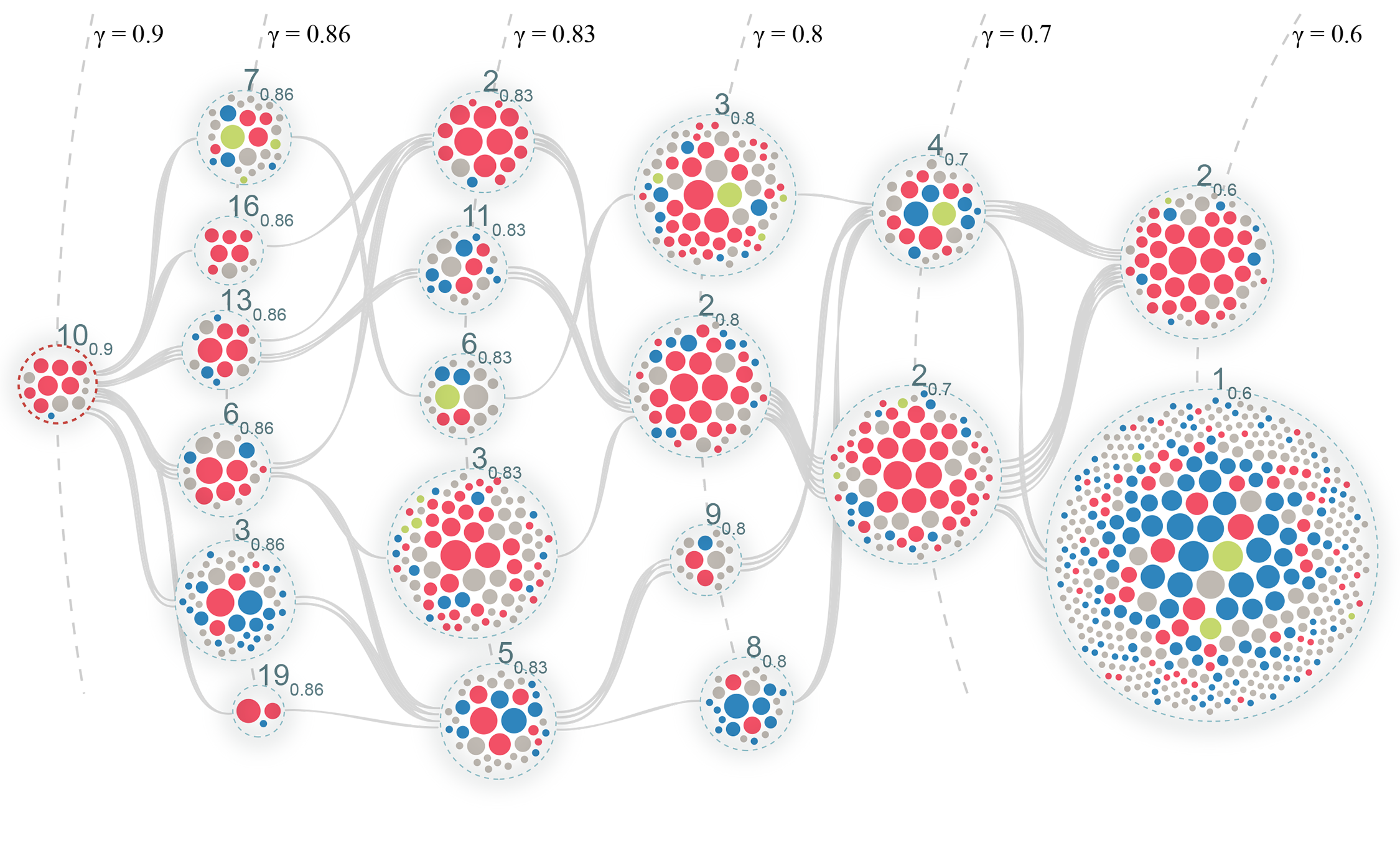

Supplement: S7 Fig — Color codes represent their dominant chromatin types. Red domains are enriched in A/B/C states, blue domains in the D-group, green domains in a combination of A/B/C and D-group states, and gray domains show no significant enrichment (p-value set at 0.025). The domain content of communities varies, with some dominated by active chromatin types and others suppressed, similar to the well-known A/B compartmentalization. As resolution increases from γ = 0.6 to γ = 0.9, domains reorganize into a community dominated by A/B/C chromatin. At intermediate scales, domains preferentially exchange between structural scales while maintaining biological similarity. (TIF) [file pcbi.1011185.s007.tif]

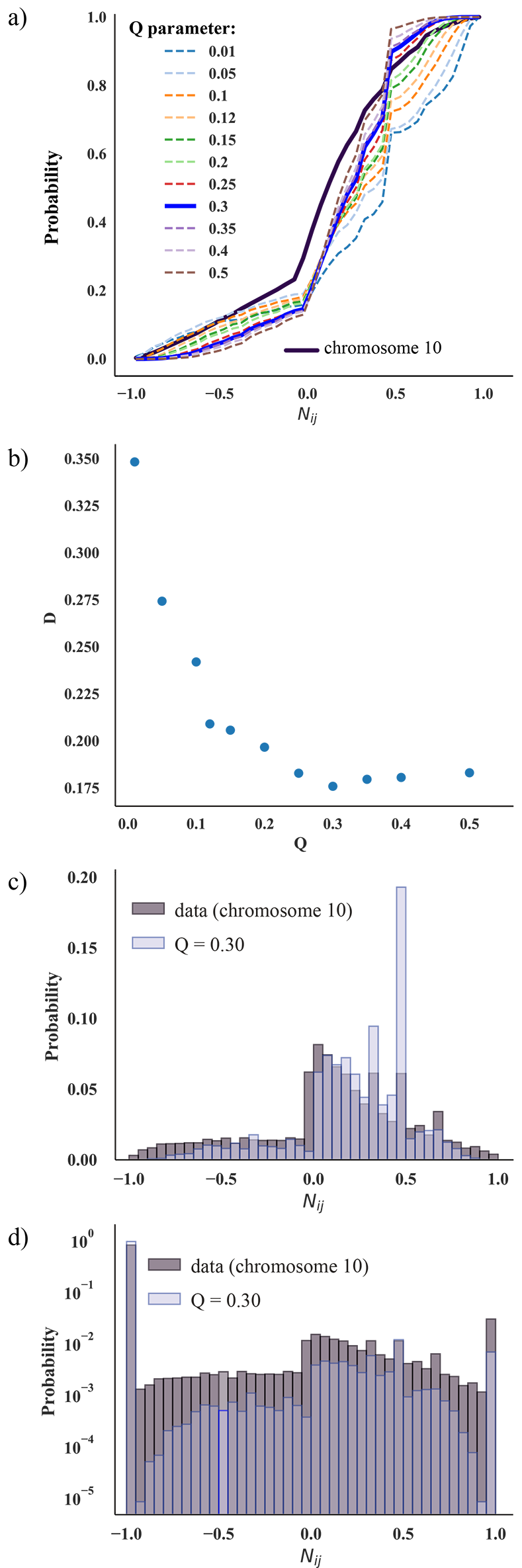

Supplement: S8 Fig — (a) CDF for nestedness score for the real data (black thick line) and models associated with varying Q. In all CDFs, the extreme Nij = ±1 values are excluded. (b) Kolmogorov-Smirnov distance, D, versus the reshuffling parameter Q. We find the minimal distance when Q = 0.3. This represents the optimal Qrmopt.. (c) Normalized Nij distributions for chromosome 10 and Q = 0.3 with Nij = ±1 removed from the plot. (d) Same as panel c), but including all Nij counts. We use log-scaled axes to fit the ±1 peaks. (TIF) [file pcbi.1011185.s008.tif]

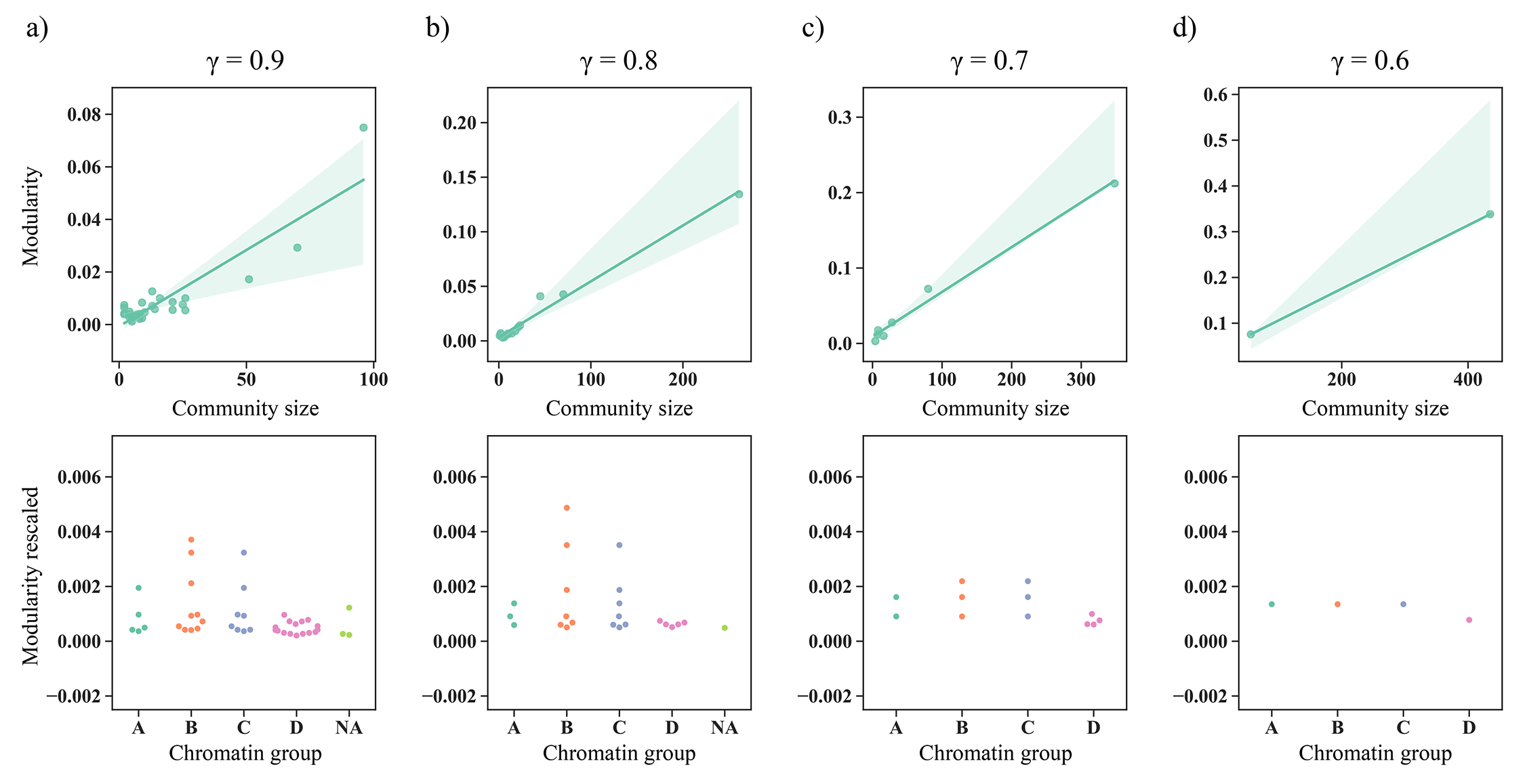

Supplement: S9 Fig — The top panels show a linear regression fit between community size (number of domains) and modularity (Eq (1), main text); We observe a nearly linear relationship. The bottom panels show the community modularity rescaled with the community size. We define the A—D chromatin groups in Methods: Chromatin states and folds of enrichment (main text). The ‘NA’ group represents communities that are not enriched in any chromatin group. (TIF) [file pcbi.1011185.s009.tif]

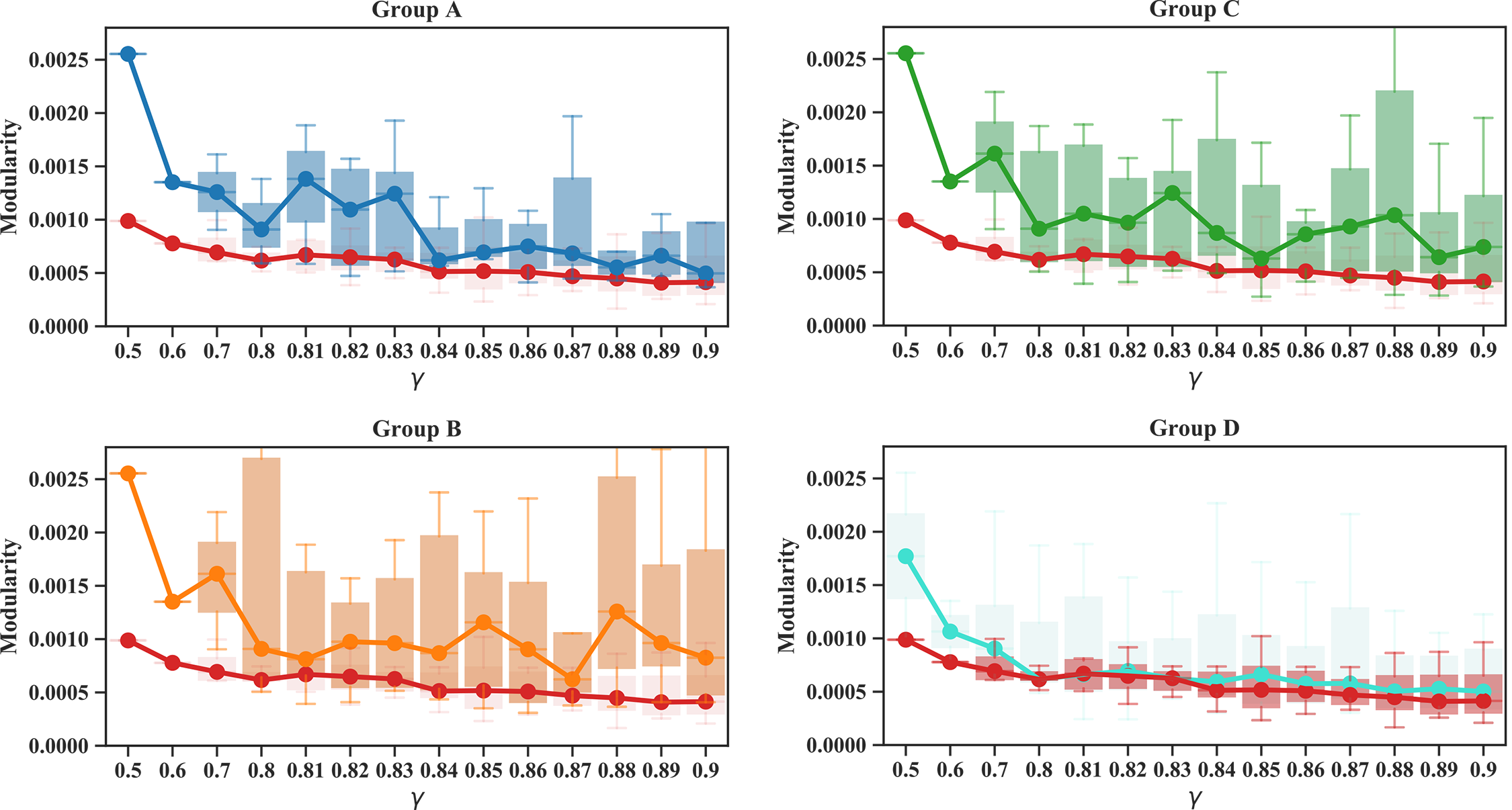

Supplement: S10 Fig — The top four plots visualize community modularity as bar plots, where values of median modularity are connected across γ. In bar plots, we compare groups A (blue), B (yellow), and C (green) with group D (red bar plot). However, the D group we compare with the modularity of all communities (light blue plot). (TIF) [file pcbi.1011185.s010.tif]
