# Supplementary material for: Mapping the semi-nested community structure of 3D chromosome contact networks
Source: PLoS Comput Biol. 2023 Jul 11;19(7):e1011185. doi: 10.1371/journal.pcbi.1011185 (PMC10361492; doi:10.1371/journal.pcbi.1011185)
Supplement: S1 Text — (DOCX) [file pcbi.1011185.s011.docx]

# Communities and domains for chromosomes 3, 5, and 22

In the main text, we show the domains for chromosome 10. In **S1 Fig**, we show the domain structure for chromosomes 3, 5, and 22. We use these domains to calculate the nestedness N_ij_ in **Fig 4** (main text).
