# Supplementary material for: Mapping the semi-nested community structure of 3D chromosome contact networks
Source: PLoS Comput Biol. 2023 Jul 11;19(7):e1011185. doi: 10.1371/journal.pcbi.1011185 (PMC10361492; doi:10.1371/journal.pcbi.1011185)
Supplement: S2 Text — (DOCX) [file pcbi.1011185.s012.docx]

# Community nestedness for chromosomes 3, 5, 10, and 22

In **Fig 4** (main text), we plot community nestedness N_ij_ over four chromosomes (3, 5, 10, and 22). **Fig 4** also shows significant versus random community overlaps. Here in **S3 Fig**, we show the data for each chromosome separately, panels (a)---(d). In addition, we outline below a specific example where we apply Eqs. (3)---(10) (main text) to calculate N_ij_ and the associated p-value in chromosome 22.

To calculate N_ij_, we start by organizing domains and communities in a two-layer bipartite graph (**S2B Fig**). One layer contains domains, and the other has all communities associated with different γ-values. Next, we connect these layers with links representing domain-community memberships. We illustrate how we map out these relationships in **S2A Fig**, where the domains form the ring, ordered according to DNA sequence, and the inner black circles indicate two communities. The links illustrate domain-community memberships.

To build the nestedness histograms, we calculate N_ij_ for every community pair, excluding pairs belonging to the same γ. As an example, we will calculate N_ij_ explicitly for communities 7_0.9_ and 4_0.7_ using the method we outline in Sec. II.B (main text).

First, we extract four numbers from the bipartite graph (**S2B Fig**):

- Number of domains: n = 93,

- Number of shared domains: k = 1

- Number of domains per community (community degree): d_i_ = 7 (4_0.7_), d_j_ = 6 (7_0.9_)

Notably, chromosome 22 has 96 domains. But we exclude three of them from the analysis (`1', `3', `96'---shown as large white circles) because they do not form any community regardless of γ. One of these domains is the centromere (domain `1').

Based on the numbers k, n, d_i_ and d_j_, Eq. (3) predicts that the community pair 7_0.9_ and 4_0.7_ share 0.45 domains under the random null hypothesis. However, the actual number is higher, k=1. To reach full nestedness, these communities should have shared six domains, which is the maximum possible overlap min(d_i_, d_j_). To reach this limit, the communities need 6 - 0.45 = 5.55 more domains than the random expectation. But it is only 1 - 0.45 = 0.55. These numbers give the nestedness N_ij_ = 0.55/5.55 = 0.099.

Next, we calculate the p-value associated with the nestedness. Using Eq. (8) and (10), we estimate that the probability of sharing at least one domain is 0.38. This value is much higher than the threshold of significance we set to 0.025. Therefore, we conclude that this overlap is insignificant.
