# Supplementary material for: Mapping the semi-nested community structure of 3D chromosome contact networks
Source: PLoS Comput Biol. 2023 Jul 11;19(7):e1011185. doi: 10.1371/journal.pcbi.1011185 (PMC10361492; doi:10.1371/journal.pcbi.1011185)
Supplement: S3 Text — (DOCX) [file pcbi.1011185.s013.docx]

# Characterization of irreducible domains and structural scales

## Characterization of irreducible domains

**Fig 2** (main text) shows the division of the chromosome into irreducible domains (top stripe). This figure highlights that domains have different sizes. To understand these sizes better, we construct a letter-value plot (**S4A Fig**), including all irreducible domains depicted along the turquoise stripe in **Fig 2** (main text). While the largest domain size is 30 Hi-C bins, the median is one Hi-C bin (100 kb); 100 kb is identical to the resolution limit of the Hi-C data. If raising γ beyond 0.9, the size distribution would have an even higher fraction of one-bin-sized domains. Therefore, to avoid over-partitioning, we select γ = 0.9 as the upper limit. This allows us to study the chromosome's 3D architecture at a granularity slightly above typical TAD sizes.

## Characterization of structural scales

In **S4B Fig**, we depict how the number of communities grows with the scale parameter γ, where the black line (and symbols) indicates the actual data (chromosome 10). We note that the number of communities grows exponentially (red line) with increasing γ. We also observe high volatility around this average growth rate for γ > 0.8. This observation motivates us to sample γ more frequently in the range 0.8 ≤ γ ≤ 0.9 as we expect significant structural rearrangements. To highlight our γ choices in **Fig 3** (main text), we mark some data points blue, leaving the others black.
