# Supplementary material for: Mapping the semi-nested community structure of 3D chromosome contact networks
Source: PLoS Comput Biol. 2023 Jul 11;19(7):e1011185. doi: 10.1371/journal.pcbi.1011185 (PMC10361492; doi:10.1371/journal.pcbi.1011185)
Supplement: S4 Text — (DOCX) [file pcbi.1011185.s014.docx]

# Nestedness distribution for specific γ-pairs

In **S5 Fig**, we show the nestedness distribution N_ij_ between associated with different γ pairs, γ_1_ and γ_2_. In each panel, we fix γ_1_ (0.6, 0.7, 0.8, or 0.9) while varying γ_2_. We illustrate the pairwise nestedness as stacked N_ij_ histograms. We observe that when γ_1_ is high, the counts are higher than random community overlap (N_ij_ ∼ 0) since more community pairs are partially nested than segregated (panels c and d).
