# Supplementary material for: Mapping the semi-nested community structure of 3D chromosome contact networks
Source: PLoS Comput Biol. 2023 Jul 11;19(7):e1011185. doi: 10.1371/journal.pcbi.1011185 (PMC10361492; doi:10.1371/journal.pcbi.1011185)
Supplement: S5 Text — (DOCX) [file pcbi.1011185.s015.docx]

# Kolmogorov-Smirnov test for nestedness and chromatin types

In the main text, we calculated community nestedness and showed the N_ij_ histograms for all chromatin type combinations (**Fig 6**). We found that communities enriched with active chromatin (types A, B, and C) are more nested than those enriched in heterochromatin (type D). In this section, we quantify this observation further by using a standard Kolmogorov-Smirnov test (KS-test) that compares pairwise N_ij_ cumulative distribution functions (CDFs) and calculates a ``distance'' (D). Specifically, we calculate such distances between the AA--DD, BB--DD, and CC--DD distributions.

**S6 Fig** presents the KS-test analysis. In panel (a), we plot the histograms for distributions AA, BB, CC, and DD (duplicated from **Fig 6**, main diagonal). In panel (b), we show the CDFs associated with these distributions and the KS-distance (D) between DD and all other CDFs. Analyzing panel (b), we observed that all CDFs start out at high values (Prob. > 0.6) due to the peak at N_ij_ = -1. This peak indicates complete segregation, which is a common feature for all four histograms. However, the DD distribution is the most segregated: the pink line exceeds all other CDFs for N_ij_ < 0.

Moreover, we note that the AA distribution (green) is the one deviating most from DD. To illustrate their maximum distance, we inserted a vertical bar in panel b for N_ij_ < 0 showing D_AA_=0.137 (p-value 8.1x10^-15^, two-sided).

Next, we analyze the positive N_ij_ range associated with nestedness. We observe that the AA--CC chromatin states are more nested than the DD group because the CDF curves grow faster for N_ij_ > 0.6 and eventually cross the pink line when N_ij_ > 0.7. When using the KS test to compare the AA, BB, and CC distributions to DD, we found that only BB and CC were significantly greater than DD. Specifically, the only values with p-value <0.05 for the one-sided test were D_BB_=0.038 and D_CC_=0.036.

Overall, we conclude that the KS test supports the notion that chromatin type is strongly associated with the nestedness of communities and that the active genome is more nested than the inactive genome. KS test also shows that the observations are statistically significant.
