# Supplementary material for: Mapping the semi-nested community structure of 3D chromosome contact networks
Source: PLoS Comput Biol. 2023 Jul 11;19(7):e1011185. doi: 10.1371/journal.pcbi.1011185 (PMC10361492; doi:10.1371/journal.pcbi.1011185)
Supplement: S6 Text — (DOCX) [file pcbi.1011185.s016.docx]

# Folding pathways and chromatin types

To better understand the biological relevance of folding pathways in **Fig 3B** in the main text, we added colors to highlight the dominant chromatin type of each irreducible domain (**S7 Fig**). This figure helps us understand whether communities consist of biologically similar domains across different γ values. As before, we used the hypergeometric test to quantify the enrichment for each chromatin group. The colors denote:

- Red: Enriched in at least one HMM state from groups A, B, or C.
- Blue: Enriched in HMM states from group D.
- Green: Enriched in A, B, or C chromatin groups and at least one HMM state from group D.
- Gray: No significant enrichment of any chromatin states (p-value = 0.025).

The figure illustrates how one node (irreducible domain) may be isolated from other nodes having the same chromatin type at some γ value but joins them at two other γ values, one lower and one higher. For example, we may see this scenario if tracking some of the red circles inside the 3D community labeled 10 (γ = 0.9), that passes through communities 3 and 19 (γ = 0.86), and 5 (γ = 0.3). These irreducible domains assemble in a complex merging-and-splitting behavior outlined above. Overall, the 3D communities tend to harbor irreducible domains having the same color, suggesting that they typically enrich domains with identical chromatin types. While we do not have enough data to suggest specific molecular mechanisms for this behavior, our data support a non-nested folding scheme.
