# Supplementary material for: Mapping the semi-nested community structure of 3D chromosome contact networks
Source: PLoS Comput Biol. 2023 Jul 11;19(7):e1011185. doi: 10.1371/journal.pcbi.1011185 (PMC10361492; doi:10.1371/journal.pcbi.1011185)
Supplement: S7 Text — (DOCX) [file pcbi.1011185.s017.docx]

# Optimal Q-parameter

Searching for the optimal Q parameter for the model described in Section D: "Modeling non-nested chromosome folding", we scan Q in the range from 0.01 to 0.5, aiming to minimize the distance between N_ij_ distribution from the real data and the model. As the distance metric, we choose the Kolmogorov-Smirnov's (KS) distance that reports the maximal difference between two cumulative distribution functions (CDF) in a two-sided statistical test. Before measuring the KS distance, we omit the extreme N_ij_ values (N_ij_ ± 1) from all distributions as they are the strongest features that bias the search for the optimal Q.

In **S8A Fig**, we plot CDF distributions for the data (chromosome 10, black thick line) and various Q parameters. All CDF plots have no counts at N_ij_ ± 1. The CDF for the optimal Q-parameter is shown as a thick blue line.

**S8B Fig** shows the largest distance between the CDF of the real data and Q. We observe that Q_opt_ = 0.3. Reshuffling domains with a probability of 30 percent at each structural scale, we obtained similar to the real data distribution around N_ij_ = 0, representing the random overlap. We visualize the CDF of our model at Q = 0.3 and the real data in **S8C and S8D Fig**.
