# Supplementary material for: Mapping the semi-nested community structure of 3D chromosome contact networks
Source: PLoS Comput Biol. 2023 Jul 11;19(7):e1011185. doi: 10.1371/journal.pcbi.1011185 (PMC10361492; doi:10.1371/journal.pcbi.1011185)
Supplement: S8 Text — (DOCX) [file pcbi.1011185.s018.docx]

# Community modularity and chromatin type

In **S9 Fig**, we show that community modularity depends on size. We use this size-dependence to rescale the modularity to facilitate comparisons. We also show that the rescaled modularity varies among chromatin types **S10 Fig**.
